# Emerging Patterns in Clinico-pathological spectrum of Oral Cancers

**Published:** 2013

**Authors:** Saadia Akram, Talat Mirza, M Aamir Mirza, Masood Qureshi

**Affiliations:** 1 Dr. Saadia Akram, Department of Pathology, Sindh Medical College, Karachi, Pakistan.; 2 Dr. Talat Mirza, Professor, Department of Pathology, Dow University of Health Sciences, Karachi, Pakistan.; 3 Dr. M Aamir Mirza, Department of Pathology, Dow University of Health Sciences, Karachi, Pakistan.; 4 Masood Qureshi, Professor, Department of Physiology, Dow University of Health Sciences, Karachi, Pakistan.

**Keywords:** Oral cancer, Oral Squamous cell carcinoma, Tobacco, Betel Quid, Betel quid substitutes

## Abstract

***Objective: ***To correlate the clinico-pathological aspects of Oral Squamous Cell Carcinoma **(OSCC)** with risk factors to determine the present status and variations in the profile.

***Methodology: ***One hundred patients of OSCC and one hundred age and sex matched controls were selected. Detailed demographic data, regarding age, gender, marital status, ethnicity, religion, socio-economic status along with habits, betel quid, tobacco chewing / smoking, alcohol and dietary habits was recorded. Detailed oral examination was carried out for the site of involvement and associated pathology. Histological grade was determined on microscopic examination of Hemotoxylin & Eosin (H&E) stained slides. One hundred age and sex matched controls were also evaluated for this study.

***Results: ***Ages of patients ranged from 25 to 80 years with mean age being 47.84 ± 12.18(SD). Maximum cases were detected in the fifth decade. Male: Female ratio was 2.8:1. Age in controls ranged from 22 -73 with male to female ratio being 3.54:1. In patients, most tumors were seen in buccal cavity (54%) followed by tongue (24%). Histologically 60% cases were well differentiated. Strong association with tobacco smoking and chewing, betel quid and its substitutes was detected, with smoking being more prevalent in males and betel quid in females. Significantly less number of controls were observed to be involved in these habits, with almost half having no such addictions.

***Conclusion:*** The present clinico-pathological status of oral cancer still emphasizes primary prevention by creating awareness against the devastating effects of tobacco use, betel quid, its substitutes and areca nut, which can go a long way in decreasing the incidence of this disfiguring and lethal condition.

## INTRODUCTION

Oral cancer is a debilitating and lethal disease with generally increasing incidence and consistently low survival rates for the past three decades. It is a cause of great concern all over the world and a major threat to public health in Pakistan, though a wide geographical variation in incidence and mortality is observed. It is the eighth most common malignancy internationally but in Pakistan it is the second commonest as per recent records of an established and well maintained cancer registry of Shaukat Khanum Memorial Hospital.^1^ It accounts for 15% of all new cancer cases in this region in comparison to 3% detected worldwide.^2^ The oral cancer incidence rates are the highest in Pakistan and India.^3^ The age standardized rates of worldwide incidence and mortality for oral cancer are 5.3 and 2.6 in males and 2.6 and 1.2 in females, respectively.^2^

Most cases have been reported in middle and older age groups but in recent years a number of studies have shown earlier age of incidence. More than 90% of cases of oral cancer are Squamous cell carcinomas (OSCC).^4^

OSCC has a multifactorial etiology with contributions of both genetic and environmental influences, suggesting an overwhelming role of the latter. Majority of the cases are attributable to separate and combined habits of tobacco use (smoked and chewed), alcohol consumption, betel quid, areca nut and betel quid substitutes. A number of recent studies are confirming the putative role of Human Papilloma Virus, especially in the western world, which still needs to be investigated in our population.^5^

Tobacco and alcohol are globally accepted and well documented strongest risk factors, most prevalent in the western countries with a multiplicative synergistic effect that has been shown in a number of studies.^6^^,^^7^ Developing Asian countries present a different scenario with greater prevalence of tobacco, betel quid, areca nut and its substitutes as major carcinogenic influences.^8^

Almost 90% of all oral cancers are caused by tobacco.^9^ Cigarettes are the most common of the various forms of smoked tobacco. Smokers have 27 fold more chances of developing OSCC in comparison with non-smokers^. ^Reduction or cessation in smoking leads to a decrease in its incidence and mortality.^10^

Nitrosamines are again the most important group of carcinogens in smoked and chewed tobacco. Susceptibility of a person depends not only on dose but also on uptake, metabolism, activation and detoxification of these substances. Chewable tobacco increases the risk of OSCC in proportion to its frequency and duration of use.^11^

In our country alcohol use is abhorred as it is not available in open market due to religious and social influences.^12^ Betel quid (Paan) chewing, on the other hand is a socially and culturally supported habit in Pakistan and rest of Central and South East Asia.^13^ Areca nut is highly addictive and chronic use leads to Sub mucus fibrosis and OSCC due to its mutagenic and genotoxic effects mediated by areca nut alkaloids and free radicals. Their easy availability, in form of cheap, attractive sachets is responsible for popularizing its use in our country in all age groups, even in primary school children.^14^

Betel quid substitutes (BQS) like gutka, pan masala, etc which are becoming popular in Indo- Pakistan are linked to a higher genotoxic and carcinogenic potential with major contributions from tobacco nitrosamines and areca nut alkaloids.^15^

Frequently accompanying poor oral hygiene with chronic irritative sepsis can also contribute to the carcinogenesis of established risk factors by elaboration of free radicals and cytokines. Nitrosamine formation may be augmented by the bacterial enzymes.

Epidemiological studies have described a role of hot and spicy foods and irritants in oral cancer development. Moreover deficiencies of vitamins A,C and E and micronutrients as selenium, folates are associated with increased risk of malignant transformation.^16^ Therefore vegetables and fruits have been shown to have a beneficial effect, though it may be attributable to vitamins found in abundance in these fruits.^7^

***Rationale: ***Numerous studies have been carried out in Western countries on etiological risk factors and their association to clinico-pathological aspects of Oral Cancer in order to determine the pathways of carcinogenesis. OSCC is much more common in Pakistan with a very high incidence and mortality and established risk factors which need to be re-evaluated due to changing lifestyle and hence risk exposure patterns.

## METHODOLOGY

This is a cross sectional study. It was carried out on 100 cases of clinically diagnosed and histopathologically proven cases of OSCC. The cases were collected from July 2009 – June 2012 from ENT ward of Civil Hospital, Karachi. Hundred age and sex matched controls were also selected from relatives and attendants accompanying the patients to correlate the etiologic risk factors. After informed consent all the patients were interviewed and examined in detail for local pathology.

The information regarding their age, gender, marital status, ethnicity, religion, socio-economic status and habits, especially betel quid, betel nut chewing, betel quid substitutes like gutka and pan masala, tobacco, naswar chewing, smoking and alcohol was recorded on a proforma. Dietary habits were also taken into account. Detailed general and local clinical examination was carried out along with condition of oral hygiene and dental status. The types of smoked and chewed products were noted but information regarding duration and frequency was subject to recall bias and variability over time.

The tissues obtained at time of biopsy or surgery were fixed in 10% neutral buffered formalin and sent to DDRRL for routine histopathological diagnosis. The representative sections were subjected to routine embedding, processing and staining with Hemotoxylin & Eosin (H&E).

The cases of OSCC were graded into Well Differentiated, Moderately and Poorly Differentiated tumors. SPSS version 16 was used for all statistical calculations.

## RESULTS

**Fig.1:**
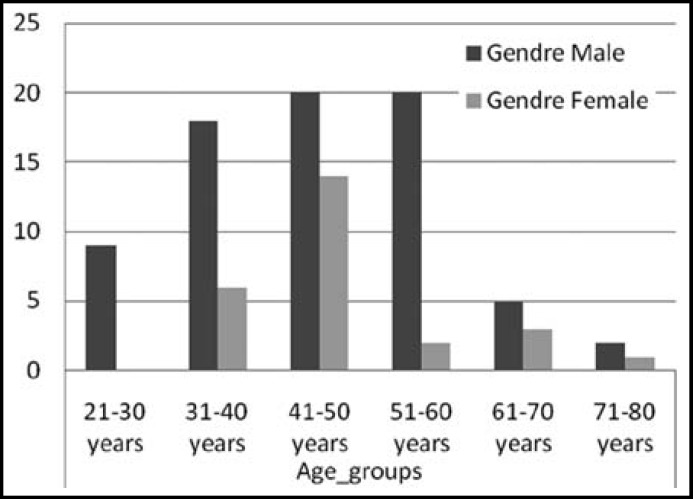
Distribution of cases according to age and gender

**Fig.2:**
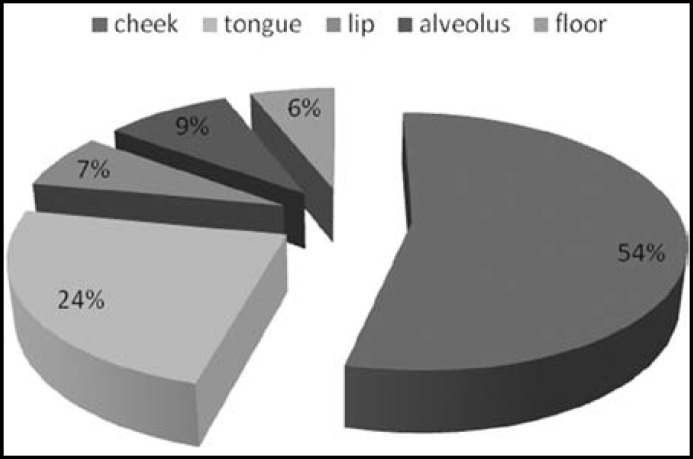
Distribution of cases according to site

**Fig.3:**
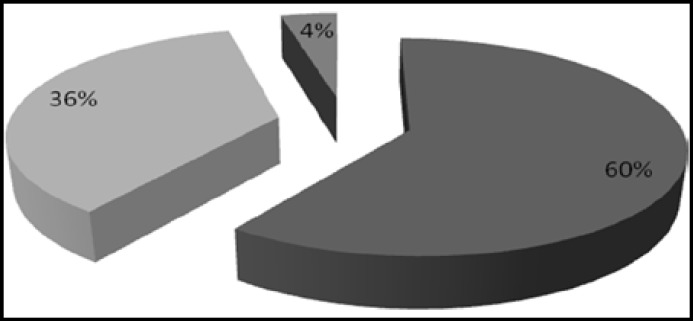
Distribution of cases according to grade

The ages of patients ranged from 25- 80 years, mean age was 47.84 +/- 12.18.Maximum number of patients was seen in the 41 – 50 years age group (Fig.1). Seventy four patients were males and 26 were females, the male **to** female ratio being 2.84:1. Among controls 78 were males and 22 were females with ages ranging from 22 -73 years. Cheek (buccal mucosa) was the most common site involved in OSCC patients in both sexes, followed by tongue and alveolar sulcus (Fig.2). Histological grading showed well differentiated malignancy in more than half the patients (Fig.3). 

**Table-I:** Comparison of Risk Factors in Cases and Controls

*Habits*		*Cases*		*Controls*
	*Males*	*Females*	*Total*	
No Habits	5	0	5	48
Tobacco Smoking	10	0	10	11
Tobacco Chewing	8	2	10	6
Betel Quid	7	20	27	15
Betel Quid Substitutes	17	3	20	10
Alcohol	1	0	1	0
Smoking with BQ	14	1	15	8
Smoking with BQ substitutes	7	0	7	2
Smoking with tobacco chewing	5	0	5	0
Total	74	26	100	100

**Table-II:** Association of Risk Factors and Habits in Cases and Controls

		*Habits*						
	*No Habits*	*Tobacco Smoking*	*Tobacco Chewing*	*Betel Quid*	*Betel Quid Substitute*	*Alcohol*	*Good fruits and veg*	*Hot spicy food*
Control	48	22	6	23	12	0	47	15
Cases	5	35	17	42	25	1	11	15
P- Value	0.000*	0.042*	0.015*	0.004*	0.018*	0.316	0.000*	1.000

* p < 0.05 - significant

Most of the patients were from low or lower middle socio-economic groups. Majority of the patients were Urdu speaking (35%), followed by Sindhis (28%), Pushtoons (15%), Punjabis (12%) and Baluchis (8%).

Information regarding risk factors revealed tobacco smoking, chewing, betel quid and its substitutes to be extensively associated with oral cancer cases. Minimal number of patients showed no habit association. Only one patient admitted to be habituated for taking alcohol. Tobacco smoking alone or in combination with other habits was dominantly found in males whereas BQ was determined as the principal risk factor in females. Betel quid use was associated with tobacco in almost all cases (Table-I). Majority of controls were not addicted to tobacco, betel quid, betel quid substitutes or alcohol. The number of controls using smoked and chewed tobacco, betel quid and betel quid substitutes was significantly less common than patients, in each group. The use of fresh fruits and vegetables was more common observation in control group.

## DISCUSSION

OSCC is found to be more common in slightly older males all over the world but newer studies are revealing its development in younger age groups.^17^ In our study the maximum numbers of cases were detected in younger age mostly affecting 41-50 years age group. This contrasts with older studies by Mirza et al^18^ showing maximum number of cases in slightly advanced age of 51-60 years. Study by Bhurgri et al^12^ in 2006 shows oral cancer in young adults conforming to the change in pattern observed in this present study.^12^^,^^18^ The reason seems to be attributable to the earlier exposure of our population to risk factors like betel quid, areca nut and its substitutes. These are popular in all age groups in both sexes due to attractive presentation, cheap prices and easy availability supported by lack of knowledge about its devastating effects. This trend for oral cancer development in younger ages is noted in the Western world as well but the cases are mostly attributable to oral HPV infection which is more prevalent there due to promiscuous sexual habits.^19^ Present study shows male predominance with male: female ratio of 2.82:1. Majority of research all over the world reveals a general male dominance.^20^

The customary risk factors as tobacco, betel quid and its substitutes show a significant association with OSCC in our subjects, conforming to the previous literature available from Indo-Pakistan Subcontinent (Table-II). Recent study by Mansoor et al also stressed the major role played by these environmental factors in oral cancer development.^13^ The most common site involved in oral cancer cases of this study was the buccal mucosa (cheek), followed by tongue which are in accordance with the risk factors rampant in our region of the world as supported by Ayaz B.^21^ The chewable forms of tobacco, betel quid, areca nut and gutka are kept in the buccal pouch for long periods and its juices are sucked in. This leads to greater exposure of the subjected mucosa to carcinogens in these habit forming substances. Western countries show tongue to be the commonly affected site in oral cancers followed by floor of mouth as their tobacco exposure is more related to smoking rather than chewing.^22^

Alcohol is a major risk factor in the Western regions^23^, but only one case of habitual alcohol intake was detected in current study. In our country alcohol intake is a taboo and it is possible that its full disclosure may have been subject to this bias and the possibility of hidden exposure in a few cases cannot be excluded.

Role of other risk factors like fruits and vegetables has been investigated which shows to have an inverse relationship with oral carcinogenesis.^17^ The present study also showed a deficient intake of fruits and vegetables in oral cancer patients. Fruits and vegetables may exert their influence indirectly through antioxidant effect of various nutrients as Vitamins A, C and E and micronutrients as iron, folic acid etc., contained therein. Independent assessment of these vitamins and micronutrients was beyond the scope of this study and requires a comprehensive epidemiological survey; however supportive evidence is still generated. Furthermore most patients evaluated belonged to the lower social strata. This could further have had a bearing on low intake of fruits and vegetables in this class which can barely afford two square meals a day for the family.

Effects of poor dentition and oral sepsis have shown equivocal results on comparing cases and controls in the present study. Detailed appraisal with microbiologic cultures for polymicrobial supragingival infections is advocated as chronic inflammation with cytokines could obviously fuel the fire for carcinogenic agents.^24^

## CONCLUSION

- Outcomes of the present study revealed differences in the risk factor exposure pattern between genders and various age groups with tendency for oral cancer development in younger ages.
- The aggressive role played by tobacco, betel quid, and areca nut and betel quid substitutes in our population reinforces the commitment for primary prevention and strong implementation of rules to curb these disguised social evils prevalent in our society at large, with immediate effect.
- There is a dire need for creating a campaign regarding awareness in all age groups, especially children against the use of these addictive carcinogenic substances, in order to bring down the incidence of this aggressive, disfiguring, and rapidly fatal condition.

## Author Contributions

SA conceived, designed, acquired and analyzed data and did writing of manuscript.

TM designed and did review, editing and final approval of manuscript.

MA did data collection and reviewed and did final approval of manuscript.

MQ did review and final approval of manuscript.

## References

[1] Cancer Registry and Clinical Data Management (CRCDM) (1994). Shaukat Khanum Memorial Cancer Hospital and Research Center (SKMCH&RC) - (www.shaukatkhanum.org.pk). Report based on cancer cases registered at SKMCH&RC from Dec.

[2] Ferlay J, Shin HR, Bray F, Forman D, Mathers C, Parkin DM (2010). Estimates of worldwide burden of cancer in 2008: GLOBOCAN 2008. Int J Cancer.

[3] Camargo CM, Voti L, Guerra M, Chapuis F, Mazuir M, Curado MP (2010). Oral cavity cancer in developed and in developing countries: Population-based incidence. Head Neck.

[4] Choi S, Myers JN (2008). Molecular pathogenesis of oral squamous cell carcinoma: implications of therapy. J Dent Res.

[5] Chaturvedi AK (2012). Epidemiology and clinical aspects of HPV in head and neck cancers. Head Neck Pathol.

[6] Zygogianni AG, Kyrgias G, Karakitsos P (2011). Oral Squamous cell cancer: early detection and the role of alcohol and smoking. Head Neck Oncol.

[7] Saman DM (2012). A review of the epidemiology of oral and pharyngeal carcinoma: update. Head Neck Oncol.

[8] Lambert R, Sauvaget C, de Camargo Cancela M, Sankaranarayanan R (2011). Epidemiologuy of cancer from the oral cavity and pharynx. Eur J Gastroenterol Hepatol.

[9] Warnakulasuriya S, Dietrich T, Bornstein MM, Peidro EC, Preshaw PM, Walter C (2010). Oral health risks of tobacco use and effects of cessation. Int Dent J.

[10] Jerjes W, Upile T, Radhi H, Petri A, Abiola J, Adams A (2012). The effect of tobacco and alcohol and their reduction/cessation on mortality in oral cancer patients: short communication. Head Neck Oncol.

[11] Jayelekshmi P, Gangadharan P, Akiba S, Koriyama C, Nair RRK (2011). Oral cavity cancer risk in relationton to tobacco chewing and biri smoking among men in Karunagappally, Kearla, India: Karunagappally cohort study. Cancer Sci.

[12] Bhurgri Y, Bhurgari A, Nishta S, Ahmed A, Pervez S, Ahmed R (2006). Pakistan Country Profile of Cancer and Cancer Control 1995-2004. J Pak Med Assoc.

[13] Khan MA, Saleem S, Shahid SM, Hameed A, Qureshi NR, Abbasi Z (2012). Prevalence of oral squamous cell carcinoma (OSCC) in relation to different chewing habits in Karachi, Pakistan. Pak J Biochem Mol Biol.

[14] Shah SM, Merchant AT, Luby SP, Chotani RA (2002). Addicted school children: prevalence and characteristics of areca nut chewing among primary school children in Karachi, Pakistan. J Paeditr Child Health.

[15] Auluck A, Hislop G, Poh C, Zhang L, Rosin MP (2009). Areca nut and betel quid chewing among South Asian immigrants to Western countries and its implications for oral cancer screening. Rural Remote Health.

[16] Lucenteforte E, Garavello W, Bosetti C, La Vecchia C (2009). Dietry factors and oral and pharyngeal cancer risk. Oral Oncol.

[17] Mafi N, Kadivar M, Hosseini N, Ahmadi S, Zare-Mirzaie A (2012). Head and Neck Squamous Cell Carcinoma in Iranian patients and risk factors in young adults: a Fifteen-Year Study. Asian Pacific J Prev.

[18] Mirza T, Alam SM, Zaidi SH, Mirza T, Rafi T (1996). Current status of Oral Cancer in Pakistan. Pak J Otolarnyngol.

[19] Brown LM, Check DP, Devesa SS (2012). Oral cavity and pharynx cancer incidence trends by subsite in the United States: Changing Gender patterns. J Oncol.

[20] Neville BW, Day TA (2002). Oral Cancer and Precancerous lesions. CA Cancer J Clin.

[21] Ayaz B, Saleem K, Azim W, Shaikh A (2011). A clinico-pathological study of oral cancers. Biomedica.

[22] Tanaka T, Ishigamori R (2011). Understanding Carcinogesesis for Fighting Oral Cancer. J Oncol.

[23] Parkin DM (2011). Cancer attributable to consumption of alcohol in UK in 2010. BJC.

[24] Khalili J (2008). Oral Cancer: Risk Factors, Prevention and Diagnostic. Exp Oncol.

